# Cytoreductive Surgery in Ovarian Cancer: Should the New Optimal Threshold Be 2.5 mm?

**DOI:** 10.3390/jcm14176094

**Published:** 2025-08-28

**Authors:** Tudor Razvan Grigorie, Gheorghe Potlog, Cosmin Verdea, Teodora Delia Chiriac, George Andrei Popescu, Dana Galieta Minca, Radu Virgil Costea, Dan Brebu, Sorin Tiberiu Alexandrescu

**Affiliations:** 1 Carol Davila University of Medicine and Pharmacy, Dionisie Lupu No. 37, Sector 2, 020021 Bucharest, Romania; razvan.grigorie@umfcd.ro (T.R.G.); teodora-delia.chiriac@drd.umfcd.ro (T.D.C.); george-andrei.popescu@drd.umfcd.ro (G.A.P.); sorin.alexandrescu@umfcd.ro (S.T.A.); 2 Department of Hepato-Bilio-Pancreatic Surgery, Emergency University Hospital Bucharest, Splaiul Independentei 169, Sector 5, 050098 Bucharest, Romania; verdea.cosmin@gmail.com; 3 Center for Digestive Diseases and Liver Transplantation, Fundeni Clinical Institute, 022328 Bucharest, Romania; g.potlog@yahoo.com; 4 Department of Public Health and Management, Dr. Leonte Anastasievici Street 1–3, Sector 5, 050463 Bucharest, Romania; 5 2nd Department of Surgery, Emergency University Hospital Bucharest, Splaiul Independentei 169, Sector 5, 050098 Bucharest, Romania; 6 10th Department of General Surgery, Victor Babes University of Medicine and Pharmacy, Piata Eftimie Murgu 2, 300041 Timisoara, Romania; brebu.dan@umft.ro

**Keywords:** optimal cytoreductive surgery, ovarian cancer, peritoneal metastases, intraperitoneal hyperthermic chemotherapy (HIPEC), survival

## Abstract

**Background/Objectives**: In patients with peritoneal metastases from ovarian cancer, current clinical guidelines recommend “optimal cytoreductive surgery (CRS)”, defined as leaving no residual tumor nodules greater than 1 cm in diameter. Of note, the 1 cm threshold is somewhat arbitrary, as even a minimal residual tumor burden may adversely impact the patient’s outcomes. The aim of the current study is to identify the independent risk factors associated with overall survival (OS) and progression-free survival (PFS) after “optimal” CRS, with a special focus on the impact of completeness of cytoreduction (defined according to Sugarbaker’s scoring system). **Methods**: This retrospective cohort study included all the patients with peritoneal metastasis from ovarian cancer who underwent “optimal CRS” (residual nodules less than 1 cm), performed by a single team. Regarding the completeness of cytoreduction (CC), the patients were divided into two groups (without residual disease or with residual nodules less than 2.5 mm (CC0/CC1), and those with residual nodules larger than 2.5 mm and less than 1 cm (“optimal” CC2)). Risk factors associated with OS and PFS were identified by univariate and multivariate analysis. **Results**: Between September 2010 and February 2025, 52 patients with a median age of 62 [53.25–66.5] years underwent “optimal” CRS. For the entire group, the median OS was 70.83 months, and the median PFS was 25.8 months. In univariate analysis, the factors associated with significantly better OS were a peritoneal cancer index (PCI) lower or equal to 10 (vs. PCI > 10; *p* = 0.025) and CC0/CC1 status (vs. “optimal” CC2; *p* = 0.004), while in multivariate analysis, the only independent factor associated with higher OS was CC0/CC1 (HR = 0.253; 95% CI: 0.092–0.696, *p* = 0.008). Regarding PFS, the only factors independently associated with higher PFS were CC0/CC1 (HR = 0.155; 95% CI: 0.046–0.527, *p* = 0.003) and no preoperative chemotherapy (HR = 0.387; 95%CI: 0.155–0.963, *p* = 0.041). **Conclusions**: To the best of our knowledge, this is the first study to reveal that in patients with peritoneal metastases from ovarian carcinoma who underwent “optimal” CRS, the only independent factor associated with both better OS and PFS was the achievement of CC0/CC1 (no residual macroscopic nodules or residual nodules less than 2.5 mm). This observation supports the notion of redefining the threshold of “optimal” cytoreduction and potentially of implementing the Sugarbaker classification of cytoreduction even in ovarian cancer.

## 1. Introduction

Among patients with newly diagnosed ovarian cancer, almost 75% have advanced stage (III or IV) disease, most of them presenting with peritoneal metastases [1]. In such stages, current guidelines recommend a combination of cytoreductive surgery (CRS) and oncologic therapy to achieve significantly increased survival and even cure [2]. If preoperative evaluation suggests that complete macroscopic resection of primary tumor and peritoneal metastases can be achieved, primary debulking surgery (PDS) followed by adjuvant chemotherapy is recommended. When complete cytoreduction cannot be initially obtained, the patients will be referred to neoadjuvant chemotherapy (NACT) prior to interval debulking surgery (IDS) [2]. The primary objective of CRS is to achieve maximal tumor debulking, ideally resulting in no gross residual disease. This approach is associated with significantly higher progression-free survival (PFS) and overall survival (OS) rates compared to palliative oncologic therapy [3,4]. Furthermore, the addition of hyperthermic intraperitoneal chemotherapy (HIPEC) has been widely discussed during the last two decades. So far, the survival benefit achieved by adding HIPEC to CRS has been proved only in responders to platinum-based neoadjuvant chemotherapy, and not in patients who underwent PDS. Nevertheless, current clinical guidelines recognize “optimal” CRS (defined by residual tumor nodules measuring less than 1 cm in diameter) as a valid and acceptable therapeutic approach [2,5,6]. However, despite the general acceptance of the <1 cm residual threshold, there is ongoing debate and criticism about whether this cutoff is sufficiently stringent. During the last 20 years, S. J. Chang, R. E. Bristow, D. S. Chi et al., and G. D. Aletti et al. have argued that defining “optimal” CRS by a residual disease threshold of less than 1 cm is somewhat arbitrary, as even minimal residual tumor burden may adversely impact the patient’s outcomes [7,8,9].

In parallel with the ongoing controversy and lack of consensus regarding the precise definition of “optimal” CRS, more refined scoring systems have been introduced to quantitatively assess surgical outcomes [10]. One notable example is Sugarbaker’s completeness of cytoreduction score (CCS), which was introduced to categorize residual disease after CRS for nongynecological malignancies. In Sugarbaker’s scoring system, a CC0 resection indicates no visible residual tumor nodules. CC1 indicates that only tiny residual nodules remain, each up to 2.5 mm in diameter. This 2.5 mm threshold was chosen because tumor nodules of that size are thought to be penetrable by intraperitoneal chemotherapy. CC2 indicates residual nodules larger than 2.5 mm but less than 2.5 cm, and CC3 indicates residual tumor nodules larger than 2.5 cm or confluence of unresectable disease [11].

Given the recognition that even “optimal” (<1 cm) residual disease is a heterogenous category, the aim of our study was to determine whether refined stratification of residual disease, based on the CC score, can reveal significant differences in clinical outcomes among patients with advanced ovarian cancer treated with “optimal” cytoreduction. The findings from this investigation will contribute to clarify if the “less than 1 cm residual nodules” benchmark is sufficient or if a more stringent threshold (such as CC0/1) should be the aspirational definition of “optimal” cytoreduction in patients with ovarian cancer and peritoneal metastases, thereby guiding surgical goals in the modern era.

In the current study, we applied Sugarbaker’s classification to a cohort of advanced ovarian cancer patients who all underwent “optimal” cytoreduction (defined as residual disease less than 1 cm). This cohort included all consecutive patients with ovarian cancer and peritoneal metastases operated by a single team. The aim of the study was to identify the independent risk factors associated with OS and PFS, with a special focus on the impact of completeness of cytoreduction (defined according to Sugarbaker’s scoring system) on the long-term outcomes of patients treated by “optimal” CRS.

## 2. Material and Methods

This is a retrospective cohort study including all patients with peritoneal metastasis from epithelial ovarian cancer who underwent “optimal” CRS performed by a single team (led by the last author of this article). Between January 2011 and October 2023, the patients underwent surgery at the Fundeni Clinical Institute Bucharest, and since November 2023, the operations were performed at the Emergency University Hospital Bucharest. The patients were selected from a prospectively maintained database. Peritoneal metastases from ovarian carcinoma were confirmed by pathologic examination. The patients with peritoneal and ovarian metastases from other malignancies (e.g., digestive cancers) were excluded. One patient with bowel obstruction due to peritoneal metastases from ovarian cancer underwent enterectomy and resection of the peritoneal nodules involving the ileum, with residual nodules larger than 1 cm (“suboptimal” CRS); this patient was also excluded from the analysis. The three patients who underwent only an open–close procedure, even after neoadjuvant chemotherapy (NACT), were also excluded.

### 2.1. Patient’s Management

Patients with newly diagnosed ovarian cancer were evaluated in a multidisciplinary meeting. Similarly, patients who developed peritoneal metastases after previous curative-intent surgery for stage I ovarian cancer, as well as those with residual peritoneal metastases after previous surgery (with or without postoperative chemotherapy) were discussed by a multidisciplinary oncologic team. All patients had preoperative chest CT scan and abdominal–pelvic CT scan or MRI to assess the loco-regional and distant extension of the disease.

Up-front surgery was recommended when preoperative imaging suggested that complete cytoreduction could be performed (primary debulking surgery—PDS). When the diagnosis of epithelial ovarian cancer had not been established preoperatively, a laparotomy was performed with fresh-frozen pathologic examination to confirm the diagnosis. Extension of the peritoneal metastases was assessed intraoperatively using the peritoneal cancer index (PCI), and residual disease was assessed by the surgeon using a completeness of cytoreduction score (CCS).

The peritoneal cancer index (PCI) is an intraoperative assessment of the extent of peritoneal disease and is calculated by the surgeon after laparotomy/laparoscopy. The PCI is calculated by giving each of the 13 abdominal regions a volume score ranging from 0 to 3 (0—no tumor, 1—tumor up to 5 mm, 2—tumor larger than 5 mm and up to 5 cm, 3—tumor larger than 5 cm). The primary tumor is excluded from lesion size assessment. The final score is the sum of the 13 regions and can range from 0 to 39 [12]. The completeness of cytoreduction was estimated according to the Sugarbaker CCS. The diameter of the largest nodule was measured using the scale from the scalpel’s handle. When it was anticipated that a CC0/CC1 could not be safely achieved (e.g., total colectomy or large enterectomies were needed), we decided to limit the operation to exploration and biopsy, referring the patient to neoadjuvant chemotherapy (NACT).

The response to NACT was assessed by an abdomino-pelvic CT scan or MRI and CT scan (usually after 3–4 cycles of NACT). When an objective response was found and the disease was considered resectable, we performed interval debulking surgery (IDS). The same strategy was followed in patients who underwent initially “suboptimal” CRS in other centers.

Although the aim of CRS was to achieve complete macroscopic resection of peritoneal nodules, sometimes this goal could not be achieved, mainly due to the residual nodules on the small bowel. In instances where extensive enterectomies for peritoneal metastases located on the small bowel surface were necessary, we ensured the preservation of a minimum of 2 m of intestinal length, although small nodules would be left on the intestine. The patient with residual nodules larger than 1 cm was excluded from analysis. Therefore, this series included only patients either without macroscopic residual disease or with residual nodules less than 1 cm located on the small bowel or colon. The nodules located on the mesentery of the preserved intestine were resected and/or electro-fulgurated.

The complexity of CRS was quantified by the Surgical Complexity Score (SCS) or Aletti score. This score assigns point(s) to each surgical maneuver, as follows: total hysterectomy with bilateral salpingo-oophorectomy—1 point; omentectomy—1 point; pelvic lymphadenectomy—1 point; para-aortic lymphadenectomy—1 point; pelvic peritoneum stripping—1 point; recto-sigmoidectomy with anastomosis—3 points; large bowel resection—2 points; diaphragm stripping/resection—2 points; splenectomy—2 points; liver resection(s)—2 points; small bowel resection(s)—1 point. The authors consider that a score ≤3 corresponds to a lower complexity operation, a score higher than 3 and lower than 8 (4–7) is correlated to an intermediate complexity procedure, while a SCS ≥ 8 points is associated with highly complex surgical intervention [13].

### 2.2. Intraperitoneal Chemotherapy

Until 2016, our hospital did not have hyperthermic intraperitoneal chemotherapy (HIPEC) capabilities. Between 2010 and 2016, we performed normothermic intraperitoneal chemotherapy. Later, we replaced the normothermic intraperitoneal chemotherapy procedure with HIPEC. Because a clear protocol was not followed, until 2020, we performed intraperitoneal chemotherapy (normothermic or hyperthermic) mainly in patients with a PCI higher than 10, without significant comorbidities, as well as in those with recurrent disease. Since 2020, only those patients with interval debulking surgery (IDS) or recurrent peritoneal metastases who responded to preoperative platinum-based chemotherapy underwent closed-abdomen HIPEC. The chemotherapeutic agent that was used for intraperitoneal administration was cisplatin, at a dose of 75 mg/m^2^ until 2021 and a dose of 100 mg/m^2^ since 2022. Until 2022, the entire dose of chemotherapy was introduced at the start of the procedure. Starting in 2023, half of the dose of cisplatin was introduced at the start of the procedure, a quarter of the dose was introduced after 30 min, and the last quarter was delivered 60 min after starting the procedure. During the HIPEC procedure, the temperature of lavage liquid was 41 degrees Celsius. The duration of the procedure was 60 min until 2021 and 90 min thereafter. Since 2024, the HIPEC procedure has been combined with the administration of sodium thiosulfate, because there is no need for preoperative hyperhydration when sodium–thiosulfate is used to prevent cisplatin-induced nephrotoxicity in patients undergoing CRS with HIPEC [14].

### 2.3. Statistical Analysis

Categorical variables were reported as numbers (and percentages) and compared by Chi square with Yates correction (due to the small sample size). Continuous variables were reported as the median and interquartile range [IQR25–IQR75]. OS was calculated as the time span between operation and the death of the patient or the date of the last follow-up if the patient was alive at that time. PFS was calculated as the time interval between CRS and the progression of malignancy (usually detected by CT scan, MRI and/or PET/CT), or until the last follow-up (if progressive disease was not identified at that time). The OS and PFS rates were assessed with the Kaplan–Meyer method and compared between different groups by the log-rank test (in univariate analysis). All variables associated with a *p* value ≤ 0.15 in univariate analysis were included in the multivariate analysis (performed by Cox regression). Multivariate analyses was performed to assess the independent prognostic factors associated with OS and PFS. The differences are considered statistically significant if the *p*-value was lower than 0.05. All statistical analyses were performed using IBM SPSS Statistics for Windows (version 26.0, IBM, Chicago, IL, USA).

## 3. Results

Between September 2010 and February 2025, our team performed “optimal” cytoreductive surgery in 52 patients with peritoneal metastases from ovarian carcinoma, with a median age of 62 [53.25–66.5] years. Three patients had stage II disease (peritoneal metastases confined to the pelvis), 37 patients had stage III disease, and 6 patients had stage IV disease. An additional six patients who were initially operated on in other hospitals for non-metastatic ovarian carcinoma had recurrent ovarian cancer with peritoneal metastases at the time of presentation to our hospital. At the time of operation, the median PCI was 11.5 [3–17], with 21 patients having a PCI as high as 10 and 31 patients having a PCI higher than 10. Clinico-pathologic characteristics of the entire group are presented in Table 1.

Out of 52 patients, 35 underwent complete cytoreduction (CC0), 12 underwent CC1 cytoreduction (residual nodules less than 2.5 mm), and 5 experienced “optimal” cytoreductive surgery, with residual peritoneal nodules ranging between 2.5 and 10 mm (CC2) in their largest diameter. The distribution of PCI and the grade of cytoreduction across different stages is depicted in Table 2. Compared to stage III, in stage IV, more patients had a PCI higher than 10, but the difference was not statistically significant (*p* = 0.181; Chi square test with Yates correction). Similarly, although in stage IV there were more patients who underwent “optimal” CC2 than in stage III, the difference did not reach statistical significance (*p* = 0.153; Chi square test with Yates correction). Regarding the association between CRS and intraperitoneal chemotherapy, 20 patients underwent HIPEC, 16 had normothermic intraperitoneal chemotherapy, while 16 patients received CRS without any type of intraperitoneal chemotherapy.

### 3.1. Short-Term Outcomes

The median length of stay was 13 [11–16] days. The rate of postoperative complications was 38.46% (20/52) (Table 3); minor complications were observed in 11 patients (21.15%), while 17.31% (9/52) patients experienced major morbidity (Clavien–Dindo grade III–V). The 30-day mortality rate was 1.92% (1/52), and the 90-day mortality rate was 3.84% (2/52).

### 3.2. Long-Term Outcomes

There were two patients who passed away in the first 90 days post-surgery due to complications unrelated to their cancer, and therefore, they were excluded from the analysis of long-term outcomes.

#### 3.2.1. Overall Survival

**Figure 1:**
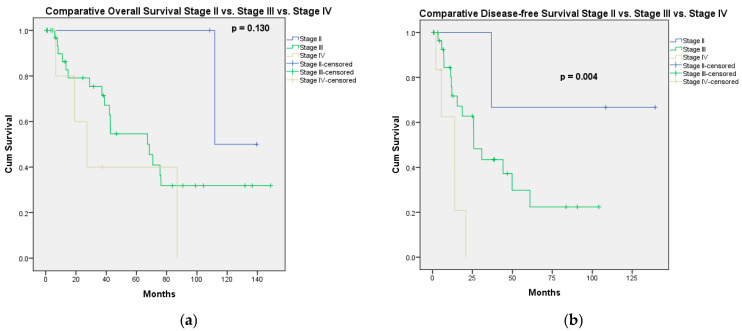
Long-term outcomes according to the stage of disease. (**a**) Overall survival according to the stage of disease; (**b**) disease-free survival according to the stage of disease.

**Figure 2:**
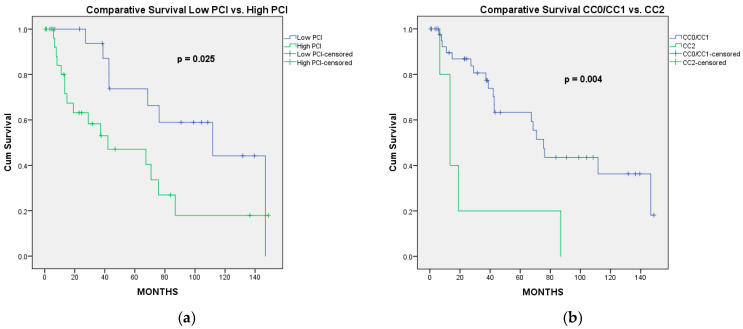
Overall survival curves of factors significantly associated with OS, according to the univariate analysis. (**a**) Low PCI (≤10) vs. high PCI (>10); (**b**) completeness of cytoreduction (CC0/CC1 vs. “optimal” CC2).

After a median follow-up of 37 [12.31–78.05] months, the 1-, 3-, 5-, and 10-year OS rates were 88.5%, 73.5%, 58.4%, and 31.2%, with a median OS of 70.83 months. According to the stage of the disease, the highest median OS (111.7 months) was observed in stage II patients. In stage III patients, the median OS was 68.5 months, while in stage IV patients, the median OS was 27.2 months (*p* = 0.130) (Figure 1a). According to the completeness of cytoreduction, the median OS of the patients who underwent CC0 was 111.7 months, while that of the patients with CC1 was 42 months (*p* = 0.104). Because the difference in OS was not statistically significant between patients with CC0 and CC1, we evaluated the OS and DFS for these patients together. Therefore, in patients receiving CC0/CC1, the median OS was 75.5 months, while in patients with “optimal” CC2 the median OS was 13.4 months (*p* = 0.004). In univariate analysis, the factors associated with significantly better OS were PCI lower or equal to 10 (vs. PCI > 10; *p* = 0.025) and CC0/CC1 status (vs. “optimal” CC2; *p* = 0.004) (Figure 2), while a low Aletti score (vs. medium/high Aletti score; *p* = 0.090) was marginally significant. In multivariate analysis, the only independent factor associated with better OS was CC0/CC1 (HR = 0.253; 95%CI: 0.092–0.696, *p* = 0.008) (Table 4).

In stage II, peritoneal dissemination is limited to the pelvis, complete cytoreduction is more frequently performed and, consequently, the prognosis of STAGE II is far better than that of higher stages. To reduce the bias introduced by these facts, a distinct survival analysis was performed, excluding the three patients with stage II. The 49 patients with advanced ovarian cancer (stage III, stage IV, or recurrence) were evaluated in a subgroup analysis. In univariate analysis, CC0/CC1 was significantly associated with better OS (*p* = 0.012), while PCI ≤ 10 was marginally associated with better OS (*p* = 0.069). In multivariate analysis, the only factor independently associated with better OS was the achievement of CC0/CC1 cytoreduction (*p* = 0.018) (Table 5).

#### 3.2.2. Progression-Free Survival

Of the 50 patients who survived more than 90 days post-surgery, 4 patients were lost to follow-up. After a median follow-up period of 19.82 [6.99–38.38] months, 21 patients were free of disease, while 25 developed progressive disease (peritoneal—15, peritoneal and hepatic—1, peritoneal and lymph nodes—1, retroperitoneal lymph nodes metastases—4, lung metastases—4). The 1-, 3-, and 5-year PFS rates were 77.2%, 40.9%, and 27.6%, respectively, with a median PFS of 25.8 months. According to the stage of disease, in stage II, the median PFS was not reached, while for stage III and stage IV, the median PFS rates were 25.9 months and 13.8 months, respectively (Figure 1b). In patients receiving CC0/CC1, the median PFS was 36.9 months, while in patients with “optimal” CC2 the median PFS was 8 months (*p* < 0.001). In univariate analysis, the factors associated with significantly better PFS were PCI lower than or equal to 10 (vs. PCI > 10; *p* = 0.028), CC0/CC1 status (vs. “optimal” CC2; *p* < 0.001), and a lower stage of disease (*p* = 0.004), while the use of preoperative chemotherapy was marginally associated with lower PFS rates compared to no preoperative chemotherapy (*p* = 0.110). In multivariate analysis, the only factors independently associated with better PFS were CC0/CC1 (HR = 0.155; 95% CI: 0.046–0.527, *p* = 0.003) and no preoperative chemotherapy (HR = 0.387; 95% CI: 0.155–0.963, *p* = 0.041) (Table 6).

In the subgroup of patients with advanced ovarian cancer (stage III, stage IV, and recurrence), univariate analysis revealed that CC0/CC1 (*p* < 0.001) and stage of disease (*p* = 0.008) were significantly associated with better PFS, while PCI ≤ 10 was marginally associated with better PFS (*p* = 0.092). In multivariate analysis, the only factor independently associated with better PFS was the achievement of CC0/CC1 cytoreduction (*p* = 0.001) (Table 7).

## 4. Discussion

In patients with peritoneal metastases from ovarian cancer, the volume of residual disease after CRS has a significant impact on PFS and OS. In 1934, Meigs suggested that the effectiveness of radiotherapy is correlated with the bulk of the residual tumor [15]. In 1975, Griffith et al. revealed that the most important factors independently associated with OS were the tumor grade and the size of the largest residual tumor mass, emphasizing the importance of adequate CRS in the treatment of these patients [16]. Over time, many authors used the term “optimal” residual disease to correlate surgical achievements with outcomes. Of note, there is no universally accepted definition of this term and, in addition, this framework may not prompt the surgeon to strive to further remove tumor tissue beyond this limit.

According to Angarita et al. who reviewed the use of the term “optimal cytoreduction” in ovarian cancer literature, 462 out of 772 publications (59.8%) used this term to describe clinical outcomes. More than 70% of these papers considered the 10 mm threshold of the largest residual nodule to differentiate between “optimal” and “suboptimal” cytoreduction [10]. In the remaining articles, the threshold was set at 20 mm (8.0%), 0 mm (6.7%), and other sizes (0.9%), while in 13.8% of these publications, the term was not defined within the text. Even recent guidelines [17,18], such as NCCN Guidelines Version 1.2025, still define residual disease less than 1 cm as “optimal” cytoreduction. However, since 2007, Winter et al. highlighted that patients with 0.1–1.0 cm residual disease had only marginally improved PFS (16.8 vs. 14.1 mo.) and OS (42.4 vs. 35 mo.) compared to patients with >1 cm residual disease for stage III ovarian cancer [19], emphasizing that maximal effort should be made to remove all gross disease since this approach offers superior survival outcomes. Similarly, the most recent randomized controlled trials (RCTs) have revealed that the higher the rate of complete cytoreduction, the longer the survival time. Thus, OVHIPEC RCT, which included 67% patients without gross residual disease and 33% with residual disease ranging between 0.1 and 1 cm, revealed that the median OS did not exceed 45 months [20]. Another RCT published by Lim et al. in 2022 reported median OS as high as 69.5 months in a group who underwent complete macroscopic resection in more than 81.5% cases (the other patients had residual disease ranging between 0.1 and 1.0 cm) [21].

An exploratory analysis of three prospectively randomized phase 3 multicenter trials that was published in 2009 revealed improved PFS and OS in patients with complete resection (*p* < 0.0001) compared to “optimal” cytoreduction (residual disease < 10 mm) or “suboptimal” CRS (residual nodules larger than 10 mm) [3]. On the other hand, although “optimal” CRS was associated with higher PFS and OS rates compared to “suboptimal” CRS, the OS difference was statistically significant only in FIGO IIIC patients. Furthermore, even in FIGO IIIC patients, the impact on OS of “optimal” CRS vs. “suboptimal” CRS was smaller (HR 0.80; 95%CI: 0.70–0.91) than those achieved by complete CRS vs. any residual tumor (HR 0.36; 95%CI: 0.31–0.42). These results suggest that the 1 cm threshold is not based on accurate evaluation of its impact on survival outcomes.

Another observation derived from RCTs is that the percentage of complete cytoreduction is directly proportional to the duration of operation. For example, in the CHORUS RCT, complete cytoreduction was achieved in only 27% of patients and “optimal cytoreduction” (0.1–1 cm) in 29%, in the context of a median length of operation of 120 (12–450, 89–160) minutes [22]. In that study, the median OS was 22.6 months in the PDS group and 24.1 months in the IDS group (*p* > 0.05). In contrast, in the RCT published by Lim et al., after a median time of operation of 405 (330.5–476.5) minutes in the control group and 525 (463.5–575) minutes in the HIPEC group, complete macroscopic resection was achieved in 87% and 81.5% of patients, respectively [21]. The median OS was 61.3 months in the control group and 69.5 months in the HIPEC group (*p* = 0.52). These results clearly show that the commitment of the surgeon (reflected by the length of operation) towards achieving complete macroscopic resection is correlated with significantly higher survival outcomes. Therefore, we believe that complete macroscopic resection should ultimately be the goal of the surgeon/oncologic gynecologist.

Complete CRS may not be feasible in all cases. The main cause for impossibility of CRS is the extensive presence of nodules on the serosa of the small bowel or colon. Their complete resection would require frequently extensive enterectomies (or total colectomy), which are not possible due to the risk of short bowel syndrome. In such instances, it is not clear what maximum diameter of the remaining nodules is associated with a meaningful survival outcome. Therefore, a more accurate, evidence-based threshold to define “optimal CRS” is mandatory.

Because the 1 cm threshold is only marginally associated with a favorable outcome, as previously mentioned, we used the Sugarbaker score (CCS) to evaluate the efficacy of CRS and categorize patients in discreet groups with potentially distinct survival outcomes. Because patients with residual nodules larger than 1 cm had unsatisfactory outcomes, we selected only those patients with residual nodules less than 10 mm in maximum diameter and divided these patients into two categories: CC0/CC1 (no residual nodules or residual nodules less than 2.5 mm) and “optimal CC2” (residual nodules between 2.5 and 10 mm). We demonstrated that “optimal CC2” is independently associated with significantly worse PFS and OS compared to CC0/CC1. Furthermore, we found that CC0/CC1 was the only independent factor associated with superior survival outcomes, both in terms of OS and PFS, in patients with advanced ovarian cancer. These data indicate that residual disease exceeding 2.5 mm, although within the traditionally accepted threshold of less than 1 cm, is associated with poorer prognosis and may not yield the same therapeutic benefit as more complete cytoreduction. This finding supports the need to refine the current criteria defining “optimal” cytoreduction, suggesting, on one hand that a 2.5 mm cutoff of residual nodules would be a more accurate threshold to define “optimal” cytoreduction and, on the other hand, that Sugarbaker’s classification is more precise to estimate the prognosis of these patients. Our observation is concordant with the definition of “optimal” cytoreduction that was used in a multi-institutional study that included 1491 patients with peritoneal metastases from epithelial high-grade serous carcinoma in the ovary, fallopian tubes, or peritoneum, operated on by surgeons who were trainers or trained within Peritoneal Surface Oncology Group International (PSOGI) units [23]. In their study, the cytoreduction was considered “optimal” when CC0 or CC1 was achieved. Due to the commitment of these surgeons to achieve such a goal, 93.2% of patients underwent CC0/CC1 surgery after a median operative time of 9 h (HIPEC being associated with 91% cases) [23]. Their survival outcomes (median OS of 58 months for the PDS group, 60 months for the IDS group, and 42 months for recurrent cases) [23] were similar to those reported by our team.

Although previous studies have revealed that CC0/CC1 is associated with improved OS and PFS rates compared to CC2/CC3 [23,24], to the best of our knowledge, this is the first study to compare the long-term outcomes achieved by CC0/CC1 with those of patients who underwent “optimal” CC2, meaning residual nodules larger than 2.5 mm and smaller than 10 mm. The OVHIPEC trial is the only study we have found to divide patients with residual nodules smaller than 10 mm into two categories: R2a—nodules ≤ 2.5 mm, and R2b—nodules larger than 2.5 mm and less than or equal to 10 mm [20]. But the authors did not compare the survival outcomes between these groups of patients.

Among other favorable prognostic factors reported by different studies, a PCI less than 10 was frequently mentioned [25,26]. According to the current study, although in univariate analysis, the extent of peritoneal metastases quantified by a PCI ≤ 10 was associated with better survival outcomes, a PCI with a threshold of 10 was not independently associated either with OS or PFS. Our results are in line with the observations of Di Giorgio et al. [27] and Eisenkop et al. [28], who found that in ovarian cancer, prognosis seems to be influenced less by initial tumor volume and more by the extent of cytoreduction achieved. One may hypothesize that our findings are due to the threshold of PCI set at 10 in the current study. Although a multicenter retrospective cohort study revealed that PCI was the strongest independent prognostic factor for both OS and PFS across all patient groups [29], several studies have examined the association between PCI and survival outcomes in patients with ovarian cancer undergoing CRS, with or without HIPEC. These studies have shown considerable variability in their findings, and no standardized PCI cutoff has been universally established, reflecting the heterogeneity across studies. Furthermore, a meta-analysis of 20 studies that assessed the prognostic value of the PCI in predicting survival in patients with advanced ovarian cancer highlighted the variability of PCI cutoff values across studies, with most using thresholds between 10 and 20. Despite this heterogeneity, a clear pattern was observed, with median survival being longer with PCI scores below any cutoff (56.7 months) and shorter with PCI scores above any cutoff (28.8 months) [30]. Furthermore, the possibility of achieving adequate cytoreduction is correlated with the extent of peritoneal spreading (quantified by PCI). The results of the current study, which indicate that in patients with advanced ovarian cancer, the completeness of cytoreduction was the only independent factor associated with OS and PFS, suggest that the ability to achieve CC0/CC1 could overcome the extent of disease (quantified by PCI or stage of disease). On the other hand, the relatively small sample size of the current study (only six patients had stage IV disease) may explain the fact that the stage of disease was not independently associated with survival outcomes. Furthermore, a different PCI cut-off may have been associated with OS and PFS. For these reasons, future larger prospective studies with more datapoints are necessary to validate (or not) the results achieved in our series.

The significantly lower PFS observed in patients who underwent preoperative chemotherapy may be explained by the fact that NACT was recommended only in patients with advanced disease, who could not benefit by CC0/CC1 at the time of the initial presentation. Because the decision to perform PDS or IDS (after NACT) was made by the same team, it is obvious that we only referred the patients that could not benefit from an adequate PDS to neoadjuvant therapy due to their more advanced peritoneal disease. Furthermore, if some peritoneal deposits disappeared after NACT, that peritoneum might be left in place at the time of IDS, representing the cause of subsequent recurrence, thus explaining the shorter PFS in patients who underwent cytoreductive surgery after NACT. Similar observations were reported in a pooled analysis of individual patient data from the EORTC 55971 and CHORUS trials, revealing that PFS was shorter after IDS compared to PDS [31]. Furthermore, Torun et al. revealed that prior chemotherapy (more than six cycles) before surgery was an independent unfavorable prognostic factor for OS in a collective series of more than 1400 patients treated with CRS and HIPEC in 11 high-volume centers [23].

### 4.1. Limitations

The present study has some limitations due to its retrospective nature and small sample size. As a retrospective analysis, this study is inherently exposed to certain limitations, such as selection bias and the possibility of incomplete and/or missing data. Moreover, the limited number of patients may affect the generalizability of the findings and reduce the statistical power to detect more subtle associations. Another limitation is the heterogeneity of the study population. Patients with peritoneal metastases from primary ovarian cancer at various stages and with recurrent ovarian cancer were analyzed together. This grouping may have introduced variability in prognosis and treatment response that could influence survival outcomes. To reduce the impact of different stages‘ prognosis, a distinct analysis was performed, considering only the patients with advanced ovarian cancer (stage III, stage IV, and peritoneal recurrence). The results of this subgroup analysis reinforced that CC0/CC1 was the only independent factor associated with OS and PFS in patients with advanced ovarian cancer who underwent “optimal” CRS. Another limitation of the current study is the long period of recruitment. Thus, throughout the 14-year time span of this study, there have been changes in the oncologic therapy used in the treatment of patients with advanced ovarian cancer (e.g., the advent of bevacizumab and PARP inhibitors during the last decade), as well as in the indications of intraperitoneal chemotherapy (e.g., the benefit of HIPEC being clearly proven only in responders to neoadjuvant platinum-based chemotherapy).

Given these considerations, the findings should be interpreted with appropriate caution. Nonetheless, they offer valuable insight and highlight the need for future prospective, larger-scale studies to validate our conclusions and to further refine the thresholds used for cytoreductive surgery in ovarian cancer.

### 4.2. Strength

Beyond these limitations, this study has some strengths. First, if the 2.5 mm threshold of residual nodules is validated in prospective trials, the findings of this study could have a major impact on clinical guidelines.

Another key strength of this study lies in the consistency of the surgical approach: all procedures were performed by the same experienced surgical team, following standardized operative protocols and techniques, aiming to achieve complete cytoreduction or at least CC1. This homogeneity minimizes variability in surgical skill and decision-making, ensuring a high level of procedural uniformity. As a result, the impact of surgical quality as a confounding factor is significantly reduced, thereby enhancing the internal validity and precision of the oncological outcomes reported. The fact that all interventions were performed by a single team presents a great methodological advantage, reducing operative variability.

## 5. Conclusions

To the best of our knowledge, this is the first study to reveal that in patients with peritoneal metastases from ovarian carcinoma who underwent “optimal” CRS, defined as residual nodules less than 10 mm, the only independent factor associated with better OS and PFS was the achievement of CC0/CC1 status according to the Sugarbaker classification of cytoreduction (no residual macroscopic nodules or residual nodules less than 2.5 mm). In patients with residual nodules larger than 2.5 mm and smaller than 10 mm (“optimal” CC2), both the risk of death and progressive disease were significantly increased compared to patients who underwent CC0/CC1. This observation raises the question of redefining the threshold of “optimal” cytoreduction, and eventually to implement the Sugarbaker classification of cytoreduction even in ovarian cancer, to achieve a better prognostic stratification of these patients and to allow for better standardization of the results achieved by CRS. If future prospective trials validate the 2.5 mm cut-off of residual nodules as a new threshold for “optimal” CRS, this study will contribute to a major change in clinical guidelines. Furthermore, setting the “optimal” CRS threshold at 2.5 mm may prompt surgeons to improve the quality of cytoreduction, which could lead to better survival outcomes.

## Figures and Tables

**Table 1:** Clinical, pathological, and treatment characteristics of the patients.

Age	62 [53.25–66.5] Years
CA-125	400 [30.5–1000] U/mL *
PCI	11.5 [3–17]
Stage	
IIA	1
IIB	2
IIIA	2
IIIB	8
IIIC	27
IVA	1
IVB	5
Recurrent peritoneal metastases	6
Preoperative chemotherapy	
Yes	17
No	35
Resected organs	
THSO	46
Omentectomy	49
Peritonectomy	
No	9
Partial	24
Complete	19
Small bowel resection	12
Colorectal resection	28
Splenectomy	11
Cholecistectomy	10
Liver resection	4
Gastrectomy	3
Cistectomy	1
Aletti score	
Low (1–3)	9
Intermediate (4–7)	24
High (≥8)	19
HIPEC	
No	32
Yes	20
Completeness of cytoreduction	
CC0	35
CC1	12
CC2 “optimal” (<10 mm.)	5
Pathologic type	
High-grade serous carcinoma	39
Low-grade serous carcinoma	8
Carcino-sarcoma	2
Borderline serous tumor with peritoneal implants	2
Mucinous cystic carcinoma	1

* Missing data for 10 patients; PCI—peritoneal cancer index, THSO—total hysterectomy with salpingo-oophorectomy, HIPEC—hyperthermic intraperitoneal chemotherapy.

**Table 2:** Distribution of PCI and CC score according to the stage of disease.

Stage	PCI		CC Score		
	≤10	>10	CC0	CC1	CC2
Stage 2	3	0	3	0	0
Stage 3	13	24	24	11	2
Stage 4	1	5	3	1	2
Recurrence	4	2	5	0	1

**Table 3:** Types of complications and causes of death.

Type of Complication	Number of Complications	Number of Death
Pleural effusion	2	
Intestinal leak	3	
Intraabdominal abscess	3	1
Wound infection	1	
Bile leak	1	
Wound hematoma	1	
Clostridium difficile colitis	3	
Parastomal abscess	1	
Pneumonia	1	
Pancreatic fistula	1	
MSOF	1	1
Stroke	1	
Urinary tract infection	1	

**Table 4:** Univariate and multivariate analyses of risk factors associated with overall survival.

	Univariate Analysis	Multivariate Analysis		
	*p* Value	HR	95% CI	*p* Value
PCI actual	0.025 *			0.121
≤10		0.482	0.192–1.212	
>10		1	–	
Preoperative chemo No Yes	0.366			
Timing of resection Primary debulking surgery Interval debulking surgery Peritoneal recurrence	0.766			
Intraperitoneal chemotherapy No Normothermic HIPEC	0.254			
Completeness of cytoreduction	0.004 *			0.008 *
CC0/CC1		0.253	0.092–0.696	
CC2		1	–	
FIGO Stage Stage II Stage III Stage IV Recurrence	0.205			
Major complications No Yes	0.345			
Aletti score Low (1–3) Medium/high (≥4)	0.090			0.490
		1	–	
		1.261	0.653–2.437	

HR—hazard ratio, CI—confidence interval, PCI—peritoneal cancer index, *—statistically significant (univariate analysis), *italic* *—statistically significant (multivariate analysis).

**Table 5:** Univariate and multivariate analyses of risk factors associated with overall survival in patients with advanced ovarian cancer.

	Univariate Analysis	Multivariate Analysis		
	*p* Value	HR	95% CI	*p*-Value
PCI actual ≤10 >10	0.069			0.767
		0.819	0.220–3.054	
		1	–	
Preoperative chemo No Yes	0.587			
Timing of resection Primary debulking surgery Interval debulking surgery Peritoneal recurrence	0.771			
Intraperitoneal chemotherapy No HIPEC Normothermic	0.128			0.371
		0.766	0.270–2.175	
		0.411	0.119–1.418	
		1	–	
Completeness of cytoreduction CC0/CC1 CC2	0.012 *			0.018 *
		0.295	0.107–0.811	
		1	–	
FIGO Stage Stage III Stage IV Recurrence	0.333			
Major complications No Yes	0.454			
Aletti score Low (1–3) Medium/high (≥4)	0.103			0.195
		0.480	0.158–1.458	
		1	–	

HR—hazard ratio, CI—confidence interval, PCI—peritoneal cancer index, *—statistically significant (univariate analysis), *italic* *—statistically significant (multivariate analysis).

**Table 6:** Univariate and multivariate analyses of risk factors associated with progression-free survival.

	Univariate Analysis	Multivariate Analysis		
	*p* Value	HR	95% CI	*p* Value
PCI actual ≤10 >10	0.028 *			0.075
		0.422	0.163–1.091	
		1	–	
Preoperative chemo No Yes	0.110			0.041 *
		0.387	0.155–0.963	
		1	1	
Timing of resection Primary debulking surgery Interval debulking surgery Peritoneal recurrence	0.479			
Intraperitoneal chemotherapy No Normothermic HIPEC	0.552			
Completeness of cytoreduction CC0/CC1 CC2	0.001 *			0.003 *
		0.155	0.046–0.527	
		1	–	
FIGO Stage Stage II Stage III Stage IV Recurrence	0.004 *			0.313
		0.422	0.039–4.565	
		1.023	0.259–4.039	
		2.969	0.629–14.008	
		1	–	
Major complications No Yes	0.806			
Aletti score Low (1–3) Medium/high (≥4)	0.180			

HR—hazard ratio, CI—confidence interval, PCI—peritoneal cancer index, *—statistically significant (univariate analysis), *italic* *—statistically significant (multivariate analysis).

**Table 7:** Univariate and multivariate analyses of risk factors associated with progression-free survival in patients with advanced ovarian cancer.

	Univariate Analysis	Multivariate Analysis		
	*p* Value	HR	95% CI	*p* Value
PCI actual ≤10 >10	0.092			0.429
		0.672	0.251—1.797	
		1	–	
Preoperative chemo No Yes	0.198			
Timing of resection Primary debulking surgery Interval debulking surgery Peritoneal recurrence	0.554			
Intraperitoneal chemotherapy No Normothermic HIPEC	0.403			
Completeness of cytoreduction CC0/CC1 CC2	0.001 *			0.001 *
		0.127	0.040—0.407	
		1	–	
FIGO Stage Stage III Stage IV Recurrence	0.008 *			0.354
		1.449	0.408–5.148	
		3.242	0.626–16.782	
		1	–	
Major complications No Yes	0.916			
Aletti score Low (1–3) Medium/high (≥4)	0.194			

HR—hazard ratio, CI—confidence interval, PCI—peritoneal cancer index, *—statistically significant (univariate analysis), *italic* *—statistically significant (multivariate analysis).

## Data Availability

The original contributions presented in this study are included in the article. Further inquiries can be directed to the corresponding authors.

## Abbreviations

The following abbreviations are used in this manuscript:

CRS	cytoreductive surgery
RCT	randomized clinical trial
PDS	primary debulking surgery
IDS	interval debulking surgery
NACT	neoadjuvant chemotherapy
HIPEC	hyperthermic intraperitoneal chemotherapy
CC	completeness of cytoreduction
PFS	progression-free survival
OS	overall survival
CT	computed tomography
MRI	magnetic resonance imaging
